# Alteration of tumour response to radiation by interleukin-2 gene transfer

**DOI:** 10.1054/bjoc.1999.1022

**Published:** 2000-02

**Authors:** J Lee, J P Moran, B M Fenton, C J Koch, J G Frelinger, P C Keng, E M Lord

**Affiliations:** Department of Microbiology and Immunology and Cancer Center Immunology Program, Department of Radiation Oncology, University of Rochester School of Medicine and Dentistry, Rochester, NY, 14642, USA; Department of Radiation Oncology, University of Pennsylvania, Philadelphia, PA, 19104, USA

**Keywords:** interleukin 2, hypoxia, murine tumours, radiatiosn

## Abstract

We have previously shown that BALB/c-derived EMT6 mammary tumours transfected with interleukin (IL)-2 have decreased hypoxia compared to parental tumours, due to increased vascularization. Since hypoxia is a critical factor in the response of tumours to radiation treatment, we compared the radiation response of IL-2-transfected tumours to that of parental EMT6 tumours. Because the IL-2 tumours have an altered host cell composition, which could affect the interpretation of radiation sensitivity as measured by clonogenic cells, we employed flow cytometric analysis to determine the proportion of tumour cells vs host cells in each tumour type. Using this approach, we were able to correct the plating efficiency based on the number of actual tumour cells derived from tumours, making the comparison of the two types of tumours possible. We also excluded the possibility that cytotoxic T-cells present in EMT6/IL-2 tumours could influence the outcome of the clonogenic cell survival assay, by demonstrating that the plating efficiency of cells derived from EMT6/IL-2 tumours remained unchanged after depletion of Thy-1^+^cells. The in vivo radiation response results demonstrated that IL-2-transfected tumours were more sensitive to radiation than parental EMT6 tumours. The hypoxic fraction of the EMT6/IL-2 tumours growing in vivo was markedly decreased relative to parental EMT6 tumours thus the increased sensitivity results from the increased vascularity we have previously observed in these tumours. These results indicate the potential therapeutic benefit of combining radiation and immunotherapy in the treatment of tumours. © 2000 Cancer Research Campaign


*British Journal of Cancer* (2000) **82**(4), 937–944
© 2000 Cancer Research Campaign
DOI: 10.1054/ bjoc.1999.1022, available online at http://www.idealibrary.com on IDEAL®

### Alteration of tumour response to radiation by interleukin-2 gene transfer

**J Lee[1,2], JP Moran[1,2], BM Fenton[3], CJ Koch[4], JG Frelinger[1,2], PC Keng[3] and EM Lord[1,2]**

[1]Department of Microbiology and Immunology and [2]Cancer Center Immunology Program; [3]Department of Radiation Oncology, University of Rochester School of Medicine and Dentistry, Rochester, NY 14642, USA; [4]Department of Radiation Oncology, University of Pennsylvania, Philadelphia, PA 19104, USA

**Summary** We have previously shown that BALB/c-derived EMT6 mammary tumours transfected with interleukin (IL)-2 have decreased hypoxia compared to parental tumours, due to increased vascularization. Since hypoxia is a critical factor in the response of tumours to radiation treatment, we compared the radiation response of IL-2-transfected tumours to that of parental EMT6 tumours. Because the IL-2 tumours have an altered host cell composition, which could affect the interpretation of radiation sensitivity as measured by clonogenic cells, we employed flow cytometric analysis to determine the proportion of tumour cells vs host cells in each tumour type. Using this approach, we were able to correct the plating efficiency based on the number of actual tumour cells derived from tumours, making the comparison of the two types of tumours possible. We also excluded the possibility that cytotoxic T-cells present in EMT6/IL-2 tumours could influence the outcome of the clonogenic cell survival assay, by demonstrating that the plating efficiency of cells derived from EMT6/IL-2 tumours remained unchanged after depletion of Thy-1$^+$ cells. The in vivo radiation response results demonstrated that IL-2-transfected tumours were more sensitive to radiation than parental EMT6 tumours. The hypoxic fraction of the EMT6/IL-2 tumours growing in vivo was markedly decreased relative to parental EMT6 tumours thus the increased sensitivity results from the increased vascularity we have previously observed in these tumours. These results indicate the potential therapeutic benefit of combining radiation and immunotherapy in the treatment of tumours. © 2000 Cancer Research Campaign

**Keywords:** interleukin 2; hypoxia; murine tumours; radiation

The microenvironment of solid tumours is defined by the interactions between malignant tumour cells and host cells, a process in which cytokines play an important role. We and others (Miller et al, 1994; McAdam et al, 1995) have utilized gene transfection techniques to manipulate tumour cells to release a given cytokine in an attempt to enhance the host immune response to tumours. In preclinical models, these cytokine gene-transduced tumour cells have been shown to generate systemic anti-tumour immunity, and in some cases to eradicate micrometastases (Fearon et al, 1990; Gansbacher et al, 1990; Colombo et al, 1991). Although these results have led to the initiation of numerous clinical trials, there are some difficulties with this approach (Jaffee and Pardoll, 1997). One problem is that in patients with large tumour burdens, the host immune system appears to be overwhelmed, and thus the response is ineffective. These results suggest that it may be necessary to employ other treatment modalities to reduce the tumour load prior to using immunotherapy.

The combination of radiotherapy and cytokine-based immunotherapy is appealing in that initial radiotherapy could be used to decrease the tumour bulk, thus allowing the immune system to be more effective. In addition, generation of anti-tumour immunity could result in long-lived memory cells that could be efficacious against metastases arising after eradication of the primary tumour. Recent reports that manipulation of cytokine expression by tumour cells can also alter their response to radiation (McBride et al, 1995; Braunschweiger et al, 1996; Syljuasen et al, 1997) provide an additional incentive for combining radiotherapy with cytokine-transduced tumour vaccines. For example, the expression of certain cytokines, such as interleukin-6 (IL-6) and interferon alpha (IFN-α), have been shown to enhance intrinsic radiosensitivity of tumour cells in vitro (McBride et al, 1995; Syljuasen et al, 1997). Data from Chiang et al (1997) showed that IL-3-transfected fibrosarcomas regressed after 25 Gy irradiation, whereas parental tumours regrew, suggesting that manipulation of the tumour response to radiation therapy might be possible by cytokine gene transfection. These results suggest that the effects of cytokine expression locally within tumours may be synergistic when used with another treatment modality and could greatly increase the overall effectiveness of treatment, although the mechanisms involved have yet to be determined.

We have previously shown that secretion of IL-2 within the tumour microenvironment dramatically decreases the tumorigenicity of EMT6, a mouse mammary tumour, and increases the infiltration of host cells, including cytotoxic T-cells (McAdam et al, 1994). In addition to the changes in the immune response, we have also shown that these IL-2-transfected EMT6 tumours have significantly decreased hypoxia compared to parental tumours using EF5, a nitroimidazole compound that binds selectively to hypoxic cells (Lee et al, 1998). Furthermore, using the fluorescent dye Hoechst 33342 as an in vivo marker of perfused vessels, combined with immunochemical staining of PECAM-1 (CD31) as a marker of tumour vasculature, we found increased vascularization in the IL-2-transfected tumours. In the current study, we

*Received 9 June 1999*
*Revised 25 August 1999*
*Accepted 25 August 1999*

*Correspondence to:* EM Lord, University of Rochester School of Medicine and Dentistry, Cancer Center, PO Box 704, 601 Elmwood Ave, Rochester, NY 14642, USA

investigated whether the expression of IL-2 by tumour cells alters their radiosensitivity. Radiation sensitivity, as based on the clonogenic cell survival assay, can be biased by the presence of non-clonogenic host cells. To eliminate this potential bias, flow cytometric analysis was used to determine the proportion of tumour cells vs host cells in each tumour, thus correcting the clonogenic data.

### MATERIALS AND METHODS

### Animals and reagents

BALB/cByJ mice were purchased from the Jackson Laboratory (Bar Harbor, ME, USA), maintained in the AAALAC approved vivarium of the University of Rochester Vivarium, and used at 2–4 months of age. Guidelines for the humane treatment of animals were followed as approved by the University Committee on Animal Resources. G-418 sulphate was obtained from Gibco Laboratories (Grand Island, NY, USA). Lipofectin was obtained from Bethesda Research Laboratories (Gaithersburg, MD, USA). Cells were cultured in EX-CELL 610 media from JRH Biologics (Lenexa, KS, USA), with 100 U penicillin, 100 μg $ml^{-1}$ streptomycin, and 2% fetal bovine serum (FBS). Paramagnetic beads (Dynabeads M-450) conjugated to sheep anti-rat-IgG were obtained from Dynal Inc. (Great Neck, NY, USA).

### Construction of the expression vector for IL-2

The murine IL-2 gene was obtained from the American Type Culture Collection and subsequently subcloned into the *Sal*I/*Bam*HI cloning site of the vector pHβ-Apr-1-neo (Gunning et al, 1987). The polymerase chain reaction was used to incorporate a *Sal*I site at the 5′ end and a *Bam*HI site at the 3′ end of the gene. The final construct contained only the sequence within the coding region (Yokota et al, 1985).

### Cell lines and transfectants

EMT6.8 is a clone of EMT6, adapted to tissue culture from a BALB/c (H-$2^d$) mammary carcinoma, supplied by Dr Sara Rockwell (Rockwell et al, 1972). EMT6.8 cells were transfected using lipofectin, according to the manufacturer's directions. At 48 h after transfection, selection of transfectants was initiated with 400 μg $ml^{-1}$ of G418; 10–14 days later, cells were cloned at limiting dilution. Clones were expanded and tested for IL-2 production. The EMT6/IL-2 clone used in these studies produced 20–26 U $ml^{-1}$ of IL-2 after 48 h of culture of $10^5$ cells $ml^{-1}$ (McAdam et al, 1994).

### Tumour growth in vivo

Tumours were initiated with $2 \times 10^5$ cells for EMT6.8, or $10^6$ cells for EMT6/IL-2. The higher cell number for the EMT6/IL-2 tumours was used to compensate for their decreased tumorigenicity and to provide tumours that grew at approximately the same rate. Sixteen to 20 days later, the mice were sacrificed and the tumours were removed.

### EF5 synthesis and treatment

EF5, pentafluorinated derivative [2-(2-nitro-1H-imidazol-1-yl)-N-(2,2,3,3,3-pentafluoropropyl)acetamide] of etanidazole was synthesized by Dr R Vishnuvajjala of the National Cancer Institute (Bethesda, MD, USA). Animals were injected intravenously with 0.2 ml of 10 mM EF5 in physiological saline, 3 h before the tumour was removed for analysis.

### EF5 binding/fluorescence assay

Tumours were minced with sharp scissors, dissociated in collagenase and filtered through a fine nylon mesh to obtain single cell suspensions for assays and for flow cytometric analysis. Absence of cell clumps were verified during the haemocytometer counts. Single-cell suspensions from tumours were fixed in 4% paraformaldehyde (PFA) for 1 h at 4°C then washed three times in phosphate-buffered saline (PBS). Cells were resuspended in blocking solution containing 5% normal mouse serum (NMS) in PBS with 0.3% Tween-20 and 1.5% albumin overnight at 4°C. Following blocking, cells were pelleted and the blocking solution was aspirated away. A Cy3 (Amersham, Arlington Heights, IL, USA) conjugated anti-EF5 (ELK3-51) antibody (Koch et al, 1995) was added to the cell pellet at a concentration of 75 μg $ml^{-1}$ in PBS with 0.3% Tween-20 and 1.5% albumin. The cells were resuspended in the antibody and then placed on a rotator wheel for 6 h at 4°C. Cells were then washed three times for 40 min each in PBS with 0.3% Tween-20 on a rotator wheel and then resuspended in 1% PFA. Cells were examined on a Coulter Epics ELITE flow cytometer as described previously (Koch et al, 1995).

### Determination of the percentage of tumour-derived host cells

Single-cell suspensions from tumours were stained with 10 μg $ml^{-1}$ anti-CD45 monoclonal antibody (30-F11, PharMingen, San Diego, CA, USA) for 30 min and followed by 20 μg $ml^{-1}$ fluorescein isothiocyanate (FITC)-conjugated mouse anti-rat IgG (Jackson Immunoresearch Labs, West Grove, PA, USA) for 30 min. Cells were washed, fixed overnight in 1% paraformaldehyde with 0.01% Tween-20 and then washed in PBS with 0.5% FBS and 0.5% Tween-20. Following RNAase (90 units $ml^{-1}$ in PBS, Sigma, St Louis, MO, USA) treatment for 30 min, cells were incubated with propidium iodide (10 μg $ml^{-1}$) and stored in the dark until analysis. Cells were examined on a Coulter Epics ELITE flow cytometer.

### Isolation of tumour-derived Thy-1 expressing cells

Thy-1-expressing cells were purified from tumours as previously described (McAdam et al, 1994). Briefly, tumours were removed and dissociated in collagenase (Type I, Sigma, St Louis, MO, USA), and the tumour infiltrating lymphocytes (TILs) were purified using paramagnetic beads conjugated to anti-Thy-1.

### Irradiation

Irradiation was performed on non-anaesthetized tumour-bearing mice using a $^{137}Cs$ source operating at a dose rate of 4.0 Gy $min^{-1}$. Each mouse was confined to a plastic jig with its tumour-bearing leg extended through an opening in the side to allow the tumour to be irradiated locally. Anoxia was induced in tumours by allowing 10 min after euthanasia before irradiation. Cells dissociated from tumours were irradiated in suspension under aerobic conditions.

 © 2000 Cancer Research Campaign

### Plating efficiency assay

Clonogenic cell survival was determined immediately after irradiation. Suitable numbers of dissociated cells were plated onto 60-mm tissue culture dishes in EX-CELL 610 media supplemented with 5% FBS. Plates were incubated at 37°C for 14 days. Media was removed and the cells were fixed with methanol, stained with crystal violet and scored for colonies containing at least 50 cells. The plating efficiency of individual cells from tumours was calculated based on haemocytometer analysis of the number of cells plated, then corrected based on the CD45-negative tumour cell number. The survival curves were fitted by the multi-target model (Chadwick and Leenhouts, 1973).

### RESULTS

### Determination of the relative percentage of host cells vs tumour cells derived from tumours

We were interested in determining if the increased vascularization we had previously observed in EMT6/IL-2 tumours altered the sensitivity of the tumours to radiation. However, because the IL-2-transfected tumours have more host cells than parental tumours and these host cells are non-clonogenic (Howell and Koch, 1980), we were concerned that the clonogenic cell survival assays used to assess radiation sensitivity would be influenced by this difference. However, due to the difficulty in visually distinguishing between tumour cells and host cells when performing haemocytometer counts, more reliable markers were needed to make this distinction. It is well-established that many tumours contain cells with abnormal DNA content, whereas normal host cells are diploid (Badalament et al, 1987). We analysed the DNA content of EMT6 cells grown in vitro by flow cytometry after staining with propidium iodide for DNA content. Spleen cells were used as controls to verify the DNA content of diploid host immune cells. Although EMT6 cells were found to be tetraploid, the DNA staining of EMT6 cells in the G1 phase of the cell cycle overlaps that of host cells in G2 phase, which can result in a significant source of error in estimating the content of host cells in tumours (data not shown). As another marker to distinguish host cells from tumour cells, we used CD45, also known as leucocyte common antigen (LCA), which is expressed on host immune cells, but not on tumour cells (Ledbetter and Herzenberg, 1979). Figure 1A and B show the cells stained with propidium iodide and anti-CD45 antibody from in vivo EMT6 tumours and EMT6/IL-2 tumours respectively. Diploid and CD45-positive host cells (represented in box 1) can be distinguished from tetraploid and CD45-negative tumour cells (represented in box 2). By using this approach, we were able to quantify the relative percentages of host vs tumour cells. In EMT6 tumours, approximately 68% of the cells are tumour cells, whereas only 55% of the cells are tumour cells in EMT6/IL-2 tumours. For all of the survival experiments, plating efficiencies for each individual tumour were based on the percentage of tetraploid, CD45-positive tumour cells present in the cell suspension, as determined by this method.

### In vivo radiation response of parental and IL-2 expressing EMT6 tumours

In order to determine if the production of IL-2 in situ within the growing tumour altered the radiation sensitivity of the tumour cells, size-matched EMT6 and EMT6/IL-2 tumours were irradiated in either air-breathing or euthanized mice. Cell survival of the irradiated tumours was determined by an in vitro plating efficiency assay. The relative radiation resistances of the different tumours are shown in Figure 2. The fraction of hypoxic cells was estimated

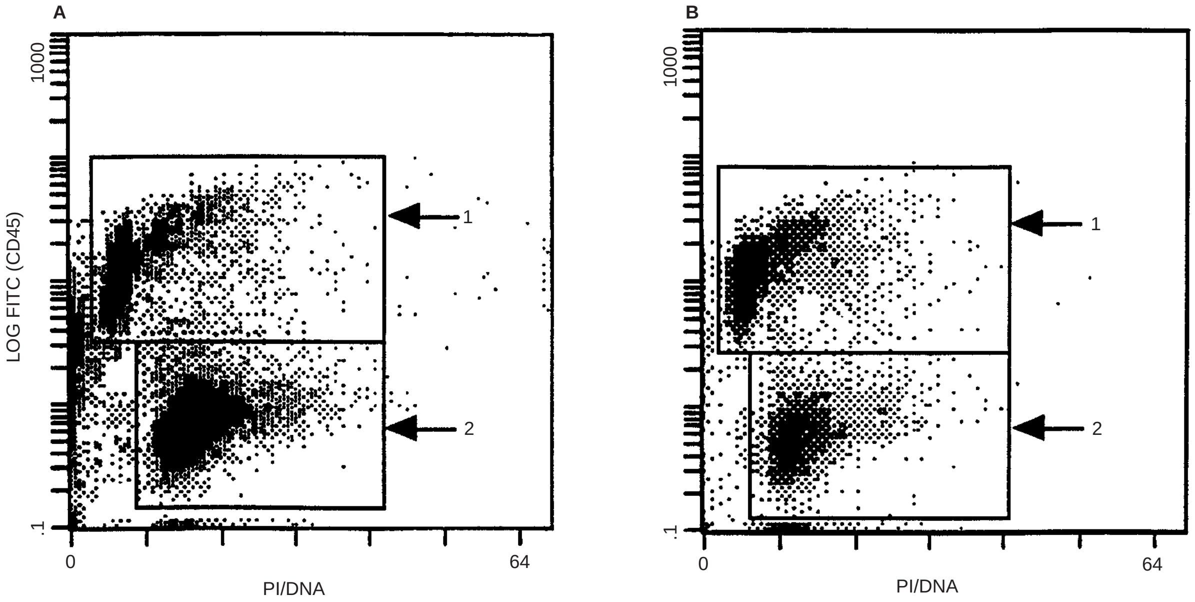

**Figure 1** Flow cytometric analysis of EMT6 (**A**) and EMT6/IL-2 (**B**) tumours to determine the relative percentage of host cells vs tumour cells. Cells derived from tumours were stained for CD45 (leucocyte common antigen) followed by staining with propidium iodide for analysis of DNA content. Box 1 represents diploid and CD45-positive host cells. Box 2 represents tetraploid and CD45-negative tumour cells. This is a representative histogram from one of the seven experiments performed. A total of 25 tumours of each type were analysed. In EMT6 tumours the percentage (mean ± s.e.) of tumour cells was 68.1% ± 1.1, whereas for EMT6/IL-2 tumours the percentage of tumour cells was 55.1% ± 1.6. The difference in these percentages were analysed by the Student's *t*-test and were significant at $P < 0.0001$

© 2000 Cancer Research Campaign

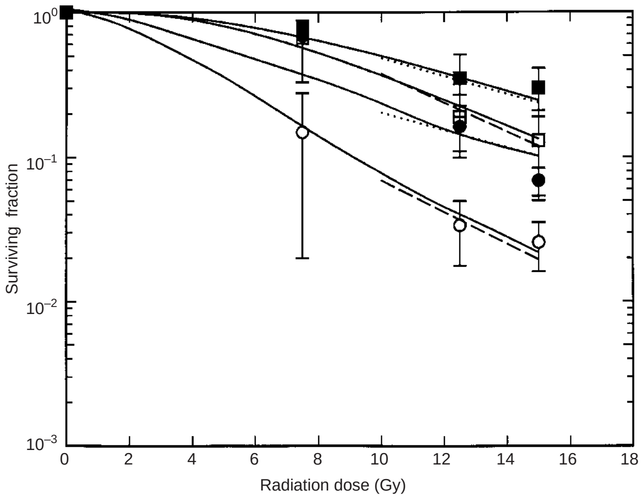

**Figure 2** Surviving fraction of tumours after irradiation in vivo. ■, EMT6 tumours irradiated in euthanized animals; □, EMT6/IL-2 tumours irradiated in euthanized animals; ●, EMT6 tumours irradiated in air-breathing animals; ○, EMT6/IL-2 tumours irradiated in air-breathing animals. Each data point represents the mean ± s.e. of 3–6 tumours assessed in three independent experiments. Lines are best fits to the individual data sets and assume multi-target kinetics.

from the ratio of the surviving fractions for air-breathing and anoxic tumour at doses of 10 Gy and higher as shown in Figure 2 (Moulder and Rockwell, 1984). The dotted and dashed lines are the best parallel lines which could be fitted for EMT6 and EMT6/IL-2 tumours. The estimated hypoxic fractions with a 95% confidence interval were 20 (14–27)% for IL-2 expressing tumours and 43 (32–53)% for parental EMT6 tumours. Thus, it is clear that the EMT6/IL-2 tumours are more radiosensitive than are the parental EMT6 tumours.

### Direct relationship between radiation survival and EF5 binding

We have previously demonstrated that EF5 binding can predict for radiation resistance in rat (Evans et al, 1996) and mouse tumours (Lee et al, 1996). To further evaluate the role of hypoxia in the increased radiosensitivity observed in the EMT6/IL-2 tumours, we compared binding of the 2-nitroimidazole drug, EF5, to the plating efficiency of cells from individual EMT6 and EMT6/IL-2 tumours after irradiation (12.5 or 15 Gy) in air-breathing mice. EF5 binding to tumour cells was evaluated by flow cytometry, and the ratio of the mean fluorescence intensity of the positively staining cells from the tumours of mice receiving EF5 compared to intensity of cells from mice that did not receive EF5 is plotted. A direct relationship between these two measurements was observed (Figure 3), further supporting the importance of tumour hypoxia in the radiosensitivity differences observed between the two tumour types.

### Influence of cytotoxic T-cells on plating efficiency

We have previously demonstrated that IL-2 secreting EMT6 tumours contain greatly increased numbers of TILs, which are lytic for EMT6 cells, and in some cases comprise more than 10% of the total cells obtained from the tumours (Lee et al, 1998). Thus, another consideration was whether cytotoxic T-cells in the cell

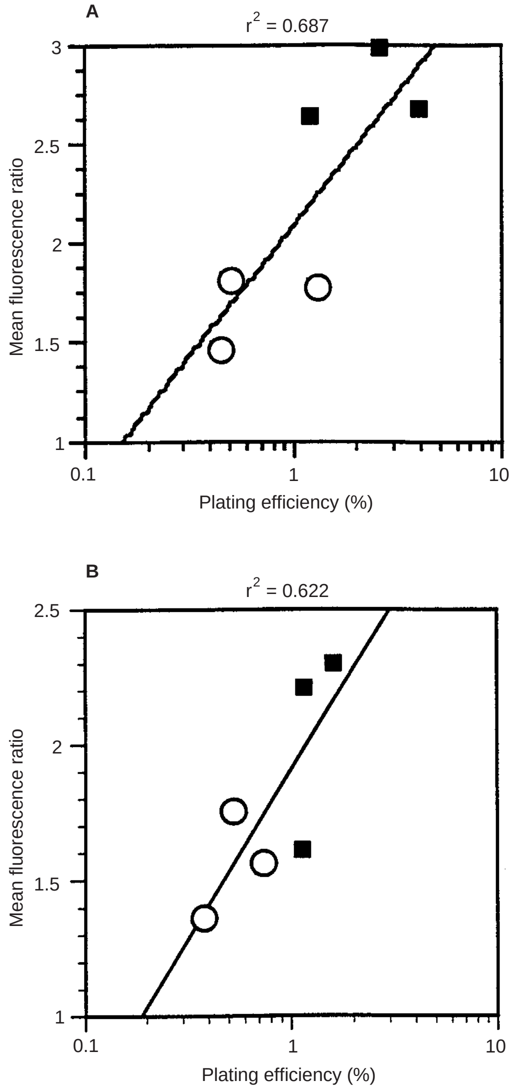

**Figure 3** Relationship between plating efficiency and the ratio of mean fluorescence (plus or minus EF5) of cells from EMT6 (■) and EMT6/IL-2 tumours (○). Tumour-bearing mice were injected with EF5 and tumour-bearing legs were exposed to 12.5 Gy (**A**) or 15 Gy (**B**). Tumours were removed and dissociated. One portion of cells was stained for EF5 binding, as described in Materials and Methods, whereas the remaining portion of cells was plated for survival analysis. Each data point represents an individual tumour.

suspensions from the EMT6/IL-2 tumours might be capable of lysing tumour cells during the plating efficiency determinations. We purified Thy-1-expressing cells from tumours using paramagnetic beads indirectly conjugated to anti-Thy-1, and compared the plating efficiency of whole tumour, Thy-1-positive cells, Thy-1-negative cells, and a recombined mixture of Thy-1-positive and -negative cells (Figure 4). As expected, the plating efficiency of EMT6 tumours was not affected by depleting Thy-1-positive cells (Figure 4A), because EMT6 tumours have only a small percentage of TILs (<3%), and these TILs exhibit minimal lysis of EMT6 cells (Lee et al, 1998). Recombining Thy-1-positive cells with Thy-1-negative cells (tumour cells and other host cells) in equal proportions also did not influence the plating efficiency. Similar results were obtained from IL-2-expressing EMT6 tumours (Figure 4B), even though these tumours have an increased number

 © 2000 Cancer Research Campaign

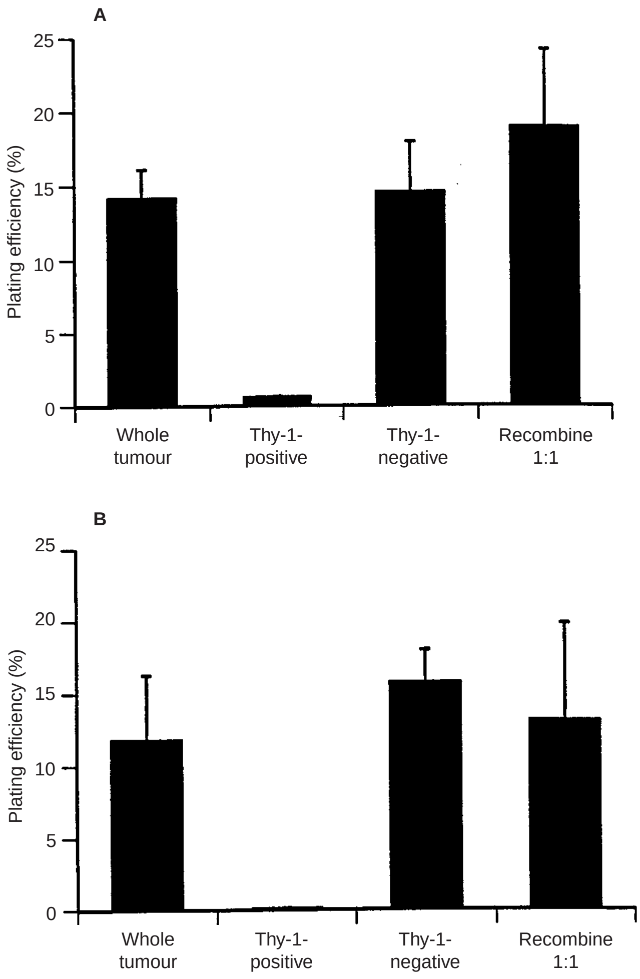

**Figure 4** The effect of TILs on the plating efficiency of cells derived from EMT6 (**A**) and EMT6/IL-2 (**B**) tumours. Thy-1-expressing cells were purified from tumours using paramagnetic beads conjugated to anti-Thy-1. The plating efficiencies of whole tumour, Thy-1-positive cells, Thy-1-negative cells, and the recombined mixture of equal numbers of Thy-1-positive and -negative cells were compared. These results are from one representative experiment of three performed. Shown are the means ± s.d. of three individual tumours of each type. As analszed by the Student's *t*-test there is no statistically significant difference between the values for the cells from the whole tumour and the Thy-1-negative cells for either tumour type.

of TILs, which lysed EMT6 cells very effectively (Lee et al, 1998). This lack of any apparent cytotoxicity of the TILs during the plating efficiency assay is probably due to the much lower cell densities in these assays compared to the standard cytotoxicity assays. These cell densities are probably not conducive to the cell-to-cell contact that is required for T-cell-mediated lysis. Therefore, in these assays we can exclude the possibility that cytotoxic T-cells present in the IL-2-expressing tumours influence the clonogenic assay.

### Intrinsic radiation sensitivity of EMT6/IL-2 cells

An additional consideration was the possibility of intrinsic differences in the radiosensitivity of EMT6/IL-2 cells and EMT6 cells. Such differences could be due to the presence of the IL-2 gene or the fact that a clone of the original cells was selected during the transfection procedure. To assess this possibility, EMT6 and EMT6/IL-2 cells growing in vitro were irradiated at various doses and survival determined. Indeed, the EMT6/IL-2 line was found to have an increased in vitro sensitivity to radiation (Figure 5). However, the survival of EMT6/Neo, transfected with the neomycin gene only, was similar to EMT6 cells, suggesting that insertion of the control vector does not affect radiosensitivity. Studies by others have suggested that altered cell proliferation and cell cycle distribution are likely to impact intrinsic radiosensitivity (Chang and Keng, 1987; Hill, 1986; Kwok and Sutherland, 1992). However, we found no differences in either growth rate or the cell cycle distribution between EMT6 and EMT6/IL-2 cells (Table 1).

To determine whether the difference in intrinsic radiosensitivity between EMT6 and EMT6/IL-2 cells also existed in tumours growing in vivo, cells were dissociated from tumours and then irradiated in suspension. Figure 6 demonstrates that cells from EMT6/IL-2 tumours are more sensitive to irradiation than those from EMT6 tumours, suggesting differences in the intrinsic radiosensitivities between the two tumour types. Each of the survival curves shown in Figure 6 was fitted using the multitarget model (Chadwick and Leenhouts, 1973), and the curves used to evaluate the oxygen enhancement ratio (OER). The OER for EMT6 tumours was 2.42 and 2.5 for EMT6/IL-2 tumours, thus the lack of difference between the two tumours also indicates some intrinsic difference in the cell lines.

### DISCUSSION

In this study, we have shown that the decreased hypoxic cell fraction observed in EMT6 mammary tumours transfected with the gene for IL-2 (Lee et al, 1998) results in increased radiation sensitivity of the tumours growing in vivo. The importance of hypoxic cells in limiting the radiation response in both animal and human tumours is well recognized (Moulder and Rockwell, 1984; Gatenby et al, 1988; Hockel et al, 1993). These results are thus in accordance with the model that tumours with decreased hypoxia (in this case due to expression of IL-2) have greater radiosensitivity. Results of several control experiments verified that the differing radiosensitivity observed was due to the microenvironmental differences present within the two types of tumours and not due to the presence of host cells confounding the in vitro plating efficiency assay. Host cells were enumerated based on their diploid DNA content and their expression of the CD45 cell surface marker thus allowing correction of the plating efficiency data. Although not necessary for all studies, it is important to make such corrections when comparing tumours having large differences in the host cell populations. This approach has the advantage of being applicable to a wide range of both experimental and human tumours. In addition, although there was a marked difference in the number and cytolytic activity of the lymphocytes present within the two types of tumours, the presence of such cytolytic host cells had no effect in the plating efficiency assay. This suggests that in most tumour systems, the presence of lytic host cells will have only minimal effects on the outcome of plating efficiency assays. Interestingly, the EMT6/IL-2 expressing line used in these experiments appeared to be inherently more radiosensitive than the parental cell line. Since there were no differences in the cell cycle and the proliferation rate, we think that the most likely explanation for the differing intrinsic radiation sensitivity between the two cell lines is clonal variation.

The response to radiation of tumours growing in vivo is influenced by numerous extrinsic factors within the tumour microenvironment,

© 2000 Cancer Research Campaign

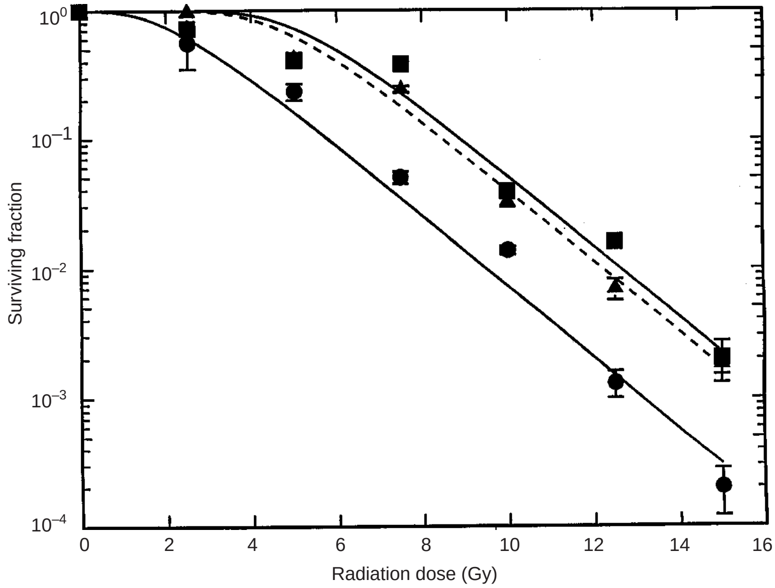

**Figure 5** The in vitro cell survival of EMT6 (■), EMT6/Neo (▲), and EMT6/IL-2 (●) cells following various doses of irradiation. The data are representative of three independent experiments. Each symbol is the mean ± s.e. of three cultures per dose. Lines are best fits to the individual data sets and assume multi-target kinetics

**Table 1** Cell cycle analysis of EMT6 and EMT6/IL-2 cells grown in vitro

| | Cells in G1 (%) | Cells in S (%) | Cells in G2 (%) |
|---|---|---|---|
| EMT6 | 42.4 | 36.4 | 21.2 |
| EMT6/IL-2 | 44.9 | 33.7 | 21.4 |

Cells were stained with propidium iodide, and DNA histograms were analysed using the multicycle program (Phoenix Flow System, San Diego, CA) on a Coulter EPICS Elite flow cytometer.

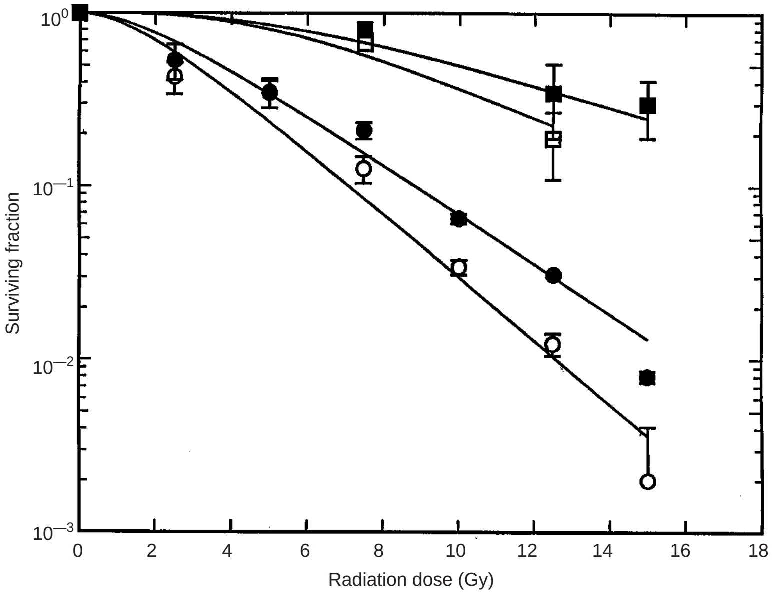

**Figure 6** Surviving fraction of anoxic tumours and tumours cells irradiated in suspension. ■, EMT6 tumours irradiated in euthanized animals (anoxia); □, EMT6/IL-2 tumours irradiated in euthanized animals; ●, EMT6 tumour-derived cells irradiated in suspension (aerobic); ○, EMT6/IL-2 tumour-derived cells irradiated in suspension. The aerated curve was generated by irradiating cells dissociated from tumours with graded radiation doses, and the hypoxia curve was generated from tumours irradiated in euthanized animals. Each data point for the anoxic curves represents the mean ± s.e. of 3–6 tumours in four independent experiments. Each data point for the aerobic curves represents the mean ± s.e. of three cultures per dose with pooled tissue from three tumours. Lines are best fits to the individual data sets and assume multi-target kinetics

 © 2000 Cancer Research Campaign in addition to the intrinsic radiosensitivity of the cell line. Tumour oxygenation is an extremely important modifier of the radiation response in the complex in vivo situation. We have previously shown that alteration of oxygenation by drug treatment or by carbogen breathing in murine KHT tumours results in an altered radiation response (Lee et al, 1996). Furthermore, in that study there was a direct relationship between the radiation response and the level of hypoxia, as measured by binding of the nitroimidazole, EF5. In the current study, where the modification of oxygenation was induced by IL-2 gene transfer, we have observed the same relationship between EF5 binding and radiosensitivity. Although we cannot exclude the possibility that the slightly altered intrinsic radiosensitivity of EMT6/IL-2 cells might also contribute to the radiosensitivity of EMT6/IL-2 tumours observed in vivo, our results strongly suggest that the increased radiosensitivity of EMT6/IL-2 tumours is mainly due to the decreased hypoxia in these tumours.

The increased vascularization and decreased hypoxia resulting in enhanced radiosensitivity that we have observed in the EMT6/IL-2 tumours provide support for combining cytokine therapy and radiotherapy to enhance tumour control. The possibility of combining these two therapy modalities has been the subject of relatively few studies. In one study it was found that combined treatment of IL-2 and TILs with local irradiation for liver metastasis in mice was more effective than single modality treatments (Cameron et al, 1990). The explanation given for this result was that local irradiation may be needed to decrease tumour bulk and to slow the tumour growth such that the rate of tumour cell lysis by TILs exceeds that of tumour growth. However, it is also possible that additional effects could be occurring. More recent reports from McBride and colleagues (McBride et al, 1995; Syljuasen et al, 1997) explored the possibility that cytokine gene transfer could be used to modify tumour radioresistance. This same group has also shown that an IL-3-transfected fibrosarcoma regressed after 25 Gy irradiation, whereas the parental tumour regrew, concluding that IL-3-transfected tumours are more sensitive than parental tumours to irradiation in vivo (Chiang et al, 1997). Interestingly, greatly increased host cell infiltration was observed in the IL-3-expressing tumours, suggesting that the regression was probably immune-mediated. In another study we have shown that IL-3 stimulates a population of antigen presenting cells (APCs) that leads to an enhanced T-cell-mediated immunity (Pulaski et al, 1996). As illustrated by the current study, IL-2 would appear to be an especially effective cytokine for combination therapy with radiation. Its ability to increase vascularization and decrease hypoxia within the tumours as previously shown (Lee et al, 1998), makes a much larger proportion of the tumour cells susceptible to radiation therapy. In addition, since the T-cells appear to infiltrate only short distances from the vessels into the tumours (Lee et al, 1998), the increased vascularization allows the T-cells stimulated by the IL-2 to access much more of the tumour.

An additional rationale for the use of IL-2 is evidence suggesting that IL-2 provides a radioprotective effect for some immune cells (Hietanen et al, 1995; Seki et al, 1995; Mor and Cohen, 1996). There has long been the concern that local irradiation might destroy the very immune cells that are likely to be the most tumour reactive, i.e. those present within the tumours and draining lymph nodes. Early studies (James et al, 1983; Gerber et al, 1989) demonstrated that IL-2 added to cultures of human T-cells made them more radioresistant. Similar effects have also been observed with natural killer cells (Hietanen et al, 1995). Similarly, work on peripheral lymphocyte populations isolated from humans (Seki et al, 1995) and rats (Mor and Cohen, 1996) has shown that IL-2 rescues T-cells from radiation-induced apoptosis, and that this was associated with induction of Bcl-2, a protein that protects cells from cell death. If indeed additional IL-2 could provide a protective effect by preventing or lessening radiation-induced apoptosis of host lymphoid cells, this would be an additional bonus for combining these modes of therapy.

Although the increase in radiosensitivity between the EMT6 and the EMT6/IL-2 tumours was not dramatic, even relatively small differences could have significant effects in a typical multifraction radiation treatment. Reoxygenation as well as initial hypoxic fraction, may also have a significant effect on tumour cure. However, these effects have not yet been studied in this system. The multiple effects observed for IL-2 on not only the immune system, but also the physical microenvironment, provides incentive to further explore the feasibility of combination therapies. A better understanding of the mechanisms involved in these cytokine-induced alterations will allow more effective use of these techniques to control malignancy.

### ACKNOWLEDGEMENTS

This work was supported by Public Health Service Grants CA28332 (to EML), CA52586 (to BMF), CA74071 (to CJK) and CA70218 (to JGF). The first two authors equally contributed to the work presented in this paper.

### REFERENCES

Badalament RA, Cibas ES, Reuter VE, Fair WR and Melamed MR (1987) Flow cytometric analysis of primary adenocarcinoma of the bladder. *J Urol* **137**: 1159–1162

Braunschweiger PG, Basrur V, Santos O, Adessa A, Houdek P and Markoe AM (1996) Radioresistance in murine solid tumors induced by interleukin-1. *Radiat Res* **145**: 150–156

Cameron RB, Spiess PJ and Rosenberg SA (1990) Synergistic antitumor activity of tumor-infiltrating lymphocytes, interleukin 2, and local tumor irradiation. Studies on the mechanism of action. *J Exp Med* **171**: 249–263

Chadwick KH and Leenhouts HP (1973) A molecular theory of cell survival. *Physics Med Biol* **18**: 78–87.

Chang AY and Keng PC (1987) Potentiation of radiation cytotoxicity by recombinant interferons, a phenomenon associated with increased blockage at the G2-M phase of the cell cycle. *Cancer Res* **47**: 4338–4341

Chiang CS, Syljuasen RG, Hong JH, Wallis A, Dougherty GJ and McBride WH (1997) Effects of IL-3 gene expression on tumor response to irradiation in vitro and in vivo. *Cancer Res* **57**: 3899–3903

Colombo MP, Ferrari G, Stoppacciaro A, Parenza M, Rodolfo M, Mavilio F and Parmiani G (1991) Granulocyte colony-stimulating factor gene transfer suppresses tumorigenicity of a murine adenocarcinoma in vivo. *J Exp Med* **173**: 889–897

Evans SM, Jenkins WT, Joiner B, Lord EM and Koch CJ (1996) 2-Nitroimidazole (EF5) binding predicts radiation resistance in individual 9L s.c. tumors. *Cancer Res* **56**: 405–411.

Fearon ER, Pardoll DM, Itaya T, Golumbek P, Levitsky HI, Simons JW, Karasuyama H, Vogelstein B and Frost P (1990) Interleukin-2 production by tumor cells bypasses T helper function in the generation of an antitumor response. *Cell* **60**: 397–403.

Gansbacher B, Zier K, Daniels B, Cronin K, Bannerji R and Gilboa E (1990) Interleukin 2 gene transfer into tumor cells abrogates tumorigenicity and induces protective immunity. *J Exp Med* **172**: 1217–1224

Gatenby RA, Kessler HB, Rosenblum JS, Coia LR, Moldofsky PJ, Hartz WH and Broder GJ (1988) Oxygen distribution in squamous cell carcinoma metastases and its relationship to outcome of radiation therapy. *Int J Radiat Oncol Biol Physics* **14**: 831–838

© 2000 Cancer Research Campaign 

Gerber M, Guichard M, Pioch Y and Dubois JB (1989) The influence of interleukin-2, feeder cells, and timing of irradiation on the radiosensitivity of human T lymphocytes assessed by the colony-forming assay. *Radiat Res* **120**: 164–176

Gunning P, Leavitt J, Muscat G, Ng SY and Kedes L (1987) A human beta-actin expression vector system directs high-level accumulation of antisense transcripts. *Proc Natl Acad Sci USA* **84**: 4831–4835

Hietanen T, Kellokumpu-Lehtinen P and Pitkanen M (1995) Action of recombinant interferons and interleukin 2 in modulating radiation effects on viability and cytotoxicity of large granular lymphocytes. *Int J Radiat Biol* **67**: 119–126

Hill RP (1986) Sensitizers and radiation dose fractionation: results and interpretations. *Int J Radiat Oncol Biol Physics* **12**: 1049–1054

Hockel M, Knoop C, Schlenger K, Vorndran B, Baussmann E, Mitze M, Knapstein PG and Vaupel P (1993) Intratumoral $pO_2$ predicts survival in advanced cancer of the uterine cervix. *Radiother Oncol* **26**: 45–50

Howell RL and Koch CJ (1980) The disaggregation, separation and identification of cells from irradiated and unirradiated EMT6 mouse tumors. *Int J Radiat Oncol Biol Physics* **6**: 311–318

Jaffee EM and Pardoll DM (1997) Considerations for the clinical development of cytokine gene-transduced tumor cell vaccines. *Methods* **12**: 143–153

James SE, Arlett CF, Green MH and Bridges BA (1983) Radiosensitivity of human T-lymphocytes proliferating in long term culture. *Int J Radiat Biol Related Studies Physics, Chem Med* **44**: 417–422

Koch CJ, Evans SM and Lord EM (1995) Oxygen dependence of cellular uptake of EF5 [2-(2-nitro-1H-imidazol-1-yl)-N-(2,2,3,3,3-pentafluoropropyl)acetamide]: analysis of drug adducts by fluroescent antibodies vs bound radioactivity. *Br J Cancer* **72**: 869–874

Kwok TT and Sutherland RM (1992) Cell cycle dependence of epidermal growth factor induced radiosensitization. *Int J Radiat Oncol Biol Physics* **22**: 525–527

Ledbetter JA and Herzenberg LA (1979) Xenogeneic monoclonal antibodies to mouse lymphoid differentiation antigens. *Immunol Rev* **47**: 63–90

Lee J, Fenton BM, Koch CJ, Frelinger JG and Lord EM (1998) Interleukin 2 expression by tumor cells alters both the immune response and the tumor microenvironment. *Cancer Res* **58**: 1478–1485

Lee J, Siemann DW, Koch CJ and Lord EM (1996) Direct relationship between radiobiological hypoxia in tumors and monoclonal antibody detection of EF5 cellular adducts. *Int J Cancer* **67**: 372–378

McAdam AJ, Felcher A, Woods ML, Pulaski BA, Hutter EK, Frelinger JG and Lord EM (1994) Transfection of transforming growth factor-beta producing tumor EMT6 with interleukin-2 elicits tumor rejection and tumor reactive cytotoxic T-lymphocytes. *J Immunother Emphasis Tumor Immunol* **15**: 155–164.

McAdam AJ, Pulaski BA, Storozynsky E, Yeh KY, Sickel JZ, Frelinger JG and Lord EM (1995) Analysis of the effect of cytokines (interleukins 2, 3, 4, and 6, granulocyte-monocyte colony-stimulating factor, and interferon-gamma) on generation of primary cytotoxic T lymphocytes against a weakly immunogenic tumor. *Cell Immunol* **165**: 183–192

McBride WH, Economou JS, Kuber N, Hong JH, Chiang CS, Syljuasen R, Dougherty ST and Dougherty GJ (1995) Modification of tumor microenvironment by cytokine gene transfer. *Acta Oncol* **34**: 447–451

Miller AR, McBride WH, Hunt K and Economou JS (1994) Cytokine-mediated gene therapy for cancer. *Ann Surg Oncol* **1**: 436–450

Mor F and Cohen IR (1996) IL-2 rescues antigen-specific T cells from radiation or dexamethasone-induced apoptosis. Correlation with induction of Bcl-2. *J Immunol* **156**: 515–522

Moulder JE and Rockwell S (1984) Hypoxic fractions of solid tumors: experimental techniques, methods of analysis, and a survey of existing data. *Int J Radiat Oncol Biol Physics* **10**: 695–712

Pulaski BA, Yeh KY, Shastri N, Maltby KM, Penney DP, Lord EM and Frelinger JG (1996) Interleukin 3 enhances cytotoxic T lymphocyte development and class I major histocompatibility complex 're-presentation' of exogenous antigen by tumor-infiltrating antigen-presenting cells. *Proc Natl Acad Sci USA* **93**: 3669–3674

Seki H, Iwai K, Kanegane H, Konno A, Ohta K, Ohta K, Yachie A, Taniguchi N and Miyawaki T (1995) Differential protective action of cytokines on radiation-induced apoptosis of peripheral lymphocyte subpopulations. *Cell Immunol* **163**: 30–36

Syljuasen RG, Belldegrun A, Tso CL, Withers HR and McBride WH (1997) Sensitization of renal carcinoma to radiation using alpha interferon (IFNA) gene transfection. *Radiat Res* **148**: 443–448

Yokota T, Arai N, Lee F, Rennick D, Mosmann T and Arai K (1985) Use of a cDNA expression vector for isolation of mouse interleukin 2 cDNA clones: expression of T-cell growth-factor activity after transfection of monkey cells. *Proc Natl Acad Sci USA* **82**: 68–72

*© 2000 Cancer Research Campaign*
